# In Vivo Biosynthesis of Inorganic Nanomaterials Using Eukaryotes—A Review

**DOI:** 10.3390/molecules25143246

**Published:** 2020-07-16

**Authors:** Ashiqur Rahman, Julia Lin, Francisco E. Jaramillo, Dennis A. Bazylinski, Clayton Jeffryes, Si Amar Dahoumane

**Affiliations:** 1 Center for Midstream Management and Science, Lamar University, Beaumont, TX 77710, USA; arahman2@lamar.com; 2 Center for Advances in Water and Air Quality & The Dan F. Smith Department of Chemical Engineering, Lamar University, Beaumont, TX 77710, USA; julia.lin9418@gmail.com (J.L.); cjeffryes@lamar.edu (C.J.); 3 School of Biological Sciences and Engineering, Yachay Tech University, Hacienda San José s/n, San Miguel de Urcuquí 100119, Ecuador; francisco.jaramillo@yachaytech.edu.ec; 4 School of Life Sciences, University of Nevada at Las Vegas, Las Vegas, NV 89154-4004, USA; dennis.bazylinski@unlv.edu

**Keywords:** nanobiotechnology, living cells, eukaryotes, nanoparticles, bioreduction, bio-applications, sustainability, post-processing, bioprocessing

## Abstract

Bionanotechnology, the use of biological resources to produce novel, valuable nanomaterials, has witnessed tremendous developments over the past two decades. This eco-friendly and sustainable approach enables the synthesis of numerous, diverse types of useful nanomaterials for many medical, commercial, and scientific applications. Countless reviews describing the biosynthesis of nanomaterials have been published. However, to the best of our knowledge, no review has been exclusively focused on the in vivo biosynthesis of inorganic nanomaterials. Therefore, the present review is dedicated to filling this gap by describing the many different facets of the in vivo biosynthesis of nanoparticles (NPs) using living eukaryotic cells and organisms—more specifically, live plants and living biomass of several species of microalgae, yeast, fungus, mammalian cells, and animals. It also highlights the strengths and weaknesses of the synthesis methodologies and the NP characteristics, bio-applications, and proposed synthesis mechanisms. This comprehensive review also brings attention to enabling a better understanding between the living organisms themselves and the synthesis conditions that allow their exploitation as nanobiotechnological production platforms as these might serve as a robust resource to boost and expand the bio-production and use of desirable, functional inorganic nanomaterials.

## 1. Introduction

Nanomaterials are defined as materials with any external dimension in the order of 1–100 nm [1]. Although there are nanomaterials with all three dimensions at the nanoscale, (nanoparticles (NPs), nanocrystals, quantum dots (QDs), etc.), most only have one or two dimensions in the nano-regime (nanofibers, nanorods, nanotubes, nanoplates, nanoribbons, nanocomposites, and nanofoams). These nano-structured materials generally possess unique physical, chemical, electrical, and magnetic properties that enable their use in numerous fields, including biomedicine, electronics, and catalysis [2].

In addition to traditional physico-chemical routes for the synthesis, sustainability, and application of inorganic NPs, biogenic nanomaterials have garnered increasing interest [3,4,5,6,7,8,9]. For example, algal cell cultures are an environmentally friendly platform for the biosynthesis of nanomaterials [10,11]. Typically, the presence of active biomolecules in algae, such as redox enzymes, can promote the in vivo or intracellular synthesis of biocompatible nanomaterials with a uniform size and shape that show pronounced antifungal, anti-bacterial, and antitumor activity [5]. In addition to intracellular enzymes, the cell surface of algae often plays a vital role in the production of nanomaterials [12]. Moreover, biopolymers present in the extracellular matrices of algal cell cultures improve the colloidal stability of some nanostructures in aqueous solutions [2,13]. However, it is imperative to note that the yield and ultimate morphology of these nanostructures depend on many factors, such as the initial cell density and chemicals’ concentrations, pH of the growth medium, incubation time, etc. [14]. The manipulation of these factors allows for a tight control over the biosynthesis process; it is also a starting point from which materials scientists could further explore these methodologies with the aim of achieving a scalable, ‘green’ NP biosynthesis process in the near future.

The synthesis of minerals by living organisms is widespread in nature with more than 60 minerals having been identified [15,16] and has become a great source of inspiration for materials scientists seeking to create novel functional materials [17,18]. This phenomenon, better known as biomineralization, has numerous examples including the production of bones and teeth by vertebrates, shells by mollusks, silica by diatoms, and magnetite crystals by salmon, birds and bees [16,19].

Biomineralization has been classified by the different types distinguished by the degree of control over the mineralization process, i.e., whether the mineral is formed actively or passively [20,21]. The process through which minerals are formed not under genetic control and are precipitated passively through cell surface charge or metabolic activity has been referred to as biologically induced mineralization [22]. The composition and properties of minerals formed through this process are strongly dependent on environmental conditions (e.g., pH, pO_2_, Eh, temperature) [23]. These minerals are generally indistinguishable from minerals formed inorganically under the same chemical conditions and are characterized by poor crystallinity, broad particle-size distributions and lack of specific crystal morphologies [22]. A good example of this process is the production of iron sulfide minerals by dissimilatory sulfate-reducing bacteria that respire with sulfate releasing sulfide ions which react with iron in the surrounding environment [22].

Biologically controlled mineralization, in contrast, is a process in which the organism exerts considerable active control over all aspects of the nucleation and mineral growth stages [24]. This process is a genetically controlled process that involves proteins encoded by specific genes that are usually part of an organic matrix responsible for the nucleation and subsequent growth of the mineral [20]. This results in mineral crystals, unlike those produced by biologically induced mineralization, that are well crystallized, show a narrow size distribution and have specific crystal morphologies [25]. One of the most well studied examples of biologically controlled mineralization is magnetite synthesis and many organisms capable of this have the ability to sense and use Earth’s geomagnetic field, a behavior known as magnetoreception [26,27]. Among these organisms capable of magnetite-based magnetoreception, the magnetotactic bacteria (MTB) have attracted special attention [28]. These unicellular prokaryotes constitute a very diverse group from phylogenetic, ecologic, and metabolic points-of-view and biomineralize, using a biologically controlled mineralization process, intracellular crystals of the magnetic minerals magnetite (Fe_3_O_4_) or greigite (Fe_3_S_4_) surrounded by a lipid bilayer membrane forming magnetosomes [25]. The composition of the mineral is dependent on the species and the concentration of dissolved oxygen or sulfide in the surrounding environment [22]. The unique chemical, physical and magnetic properties have made these magnetosomes suitable candidates for various bio-applications [29].

Biosynthesized inorganic NPs have proven useful in numerous applications in various fields due their nontoxic, eco-friendly nature and versatility [30,31,32]. For example, fungi-mediated biosynthesized NPs, such as ZnO NPs, have been successfully used in applications in the industrial, medical and agricultural sectors [33]. Biogenic TiO_2_ NPs have been used as plant nutrients [34]. Plant-mediated Pd NPs and Pt NPs have been shown to exhibit remarkable catalytic properties [35]. Moreover, it is well known that biogenic Ag NPs and Au NPs appear to be optimal (versus their non-biogenic counterparts) in certain applications in optoelectronics, cancer detection, anti-cancer drug therapeutic, antimicrobial activity, including anti-bacterial, antifungal and antiviral capabilities as well as in the biological control of insect larvae [30,31,36,37]. Furthermore, recent advancements in crosslinking of biopolymer chains have increased the stability of NPs resulting in a significant extension of their applicability and usefulness in nanomedicine, bioengineering and catalysis [30,31,38].

The present review extensively summarizes findings related exclusively to the green production of inorganic nanomaterials using living eukaryotic cells and organisms, namely live plants, living biomass of several species of microalgae, yeast, fungus, mammalian cells, and animals. Moreover, it discusses the various methodologies devised by researchers to exploit these natural, renewable resources in the field of bionanotechnology, the different inorganic nanomaterials bioproduced and their characterization. Although very scare, the present review introduces the bio-applications of the as-obtained nanomaterials whenever available. Furthermore, a special focus is given to the underlying mechanistic aspects that govern these processes as their comprehension may open new avenues for these routes and the valuable nanomaterials generated in nanoscience and nanotechnology. To that aim, several factors that affect the production and the characteristic features of the in vivo synthesized nanomaterials, such as the temperature, pH of the reaction medium, incubation time, illumination intensity, precursor ion concentration, metal cation to biomass ratio, etc. are discussed. A substantial section is dedicated to the in vivo biosynthesis of nanomaterials using microalgae that encompasses the role of the algal cell surface in NP formation, factors that affect the production and as-synthesized NP morphology along with their post-processing. Although many studies have reported cell surface involvement in the production of NPs, no comprehensive review details the effect of the cell surface on NP formation. Therefore, the present review gives special attention to understanding algal cell surface biomolecules and their involvement in NP biosynthesis. Interestingly, the current review is unique in that the post-processing of algal-synthesized nanomaterials is described. We aggregated single post-processing steps from the literature and compiled them into a comprehensive process flow diagram (PFD) for the recovery, purification, quantification, and storage of the in vivo synthesized nanomaterials. Most importantly, a similar PFD can be applied to any in vitro biosynthesis process.

## 2. In Vivo Biosynthesis of Inorganic Nanomaterials Using Plants

Extracts of plants or their parts, such as roots, leaves, flowers, bark, and seeds, have been extensively screened for their ability to promote the biosynthesis of different kinds of nanomaterials, namely metallic NPs, oxide NPs, and chalcogenide NPs. Several reviews have covered the aspects of such processes, including the methodologies used, features and diversity of as-obtained NPs, underlying mechanisms and potential bio-applications [35,39,40,41]. Numerous research papers are published weekly, adding to the already rich literature in this field. However, articles reporting on the use of whole living plants for nanomaterial synthesis are very infrequent when compared to using plants’ extracts. Among the latter, only a handful of research papers describe the use of intact chloroplasts harvested from plants to carry out the biosynthesis of Au NPs [42,43], Ag NPs [44] and even bimetallic Ag-Au alloy NPs [45].

After feeding living alfalfa sprouts with the corresponding precursors, Gardea-Torresdey demonstrated that it was possible to produce *in planta* Au NPs [46] and Ag NPs [47]. In both cases, the presence of Au NPs and Ag NPs inside the plant was confirmed using several analytical techniques. Haverkamp et al. [48] deepened this process by implementing the synthesis of trimetallic Au-Ag-Cu alloy NPs inside the living plant, *Brassica juncea*, by growing it in a soil that contained 48 mg kg^−1^ Au (0.24 mmol kg^−1^), 44 mg kg^−1^ Cu (0.67 mmol kg^−1^) and 31 mg kg^−1^ Ag (0.29 mmol kg^−1^). The as-obtained NPs varied from 5–50 nm in diameter. The alloy nature of these nanocrystals was determined using scanning transmission electron microscopy—energy dispersive X-ray spectroscopy (STEM-EDX) and X-ray diffraction (XRD). Moreover, the atomic composition of the large NPs that formed inside this plant varied a lot; these NPs were mostly made of silver (44–80%) and gold (55–20%) while copper represented up to 1%. Analyses carried out on smaller NPs revealed a similar atomic composition trend: 57% Ag, 43% Au and less than 1% Cu. Although the molar ratio of Ag to Au in these small NPs (1.33:100 Ag:Au) differs slightly from the one of the initial input (1.21:1.00 Ag:Au), the percentage of copper, in all cases, was negligible compared to the initial amount of its precursor present in the feeding soil of *B. juncea*. This indicates that the plant did not synthesize NPs with an elemental composition reflective of the stoichiometric ratio of the metal salts added to the feeding soil. Moreover, the element with the highest initial molar ratio, copper, was almost absent in the NPs. The low standard potential of Cu^2+^ (E° = 0.34 V), compared to Au^3+^ (E° = 1.52 V) and Ag^+^ (E° = 0.80 V), may explain why metallic gold and silver were observed in greater quantities in the NPs than metallic copper. Extracts of diverse plants and various plant parts, i.e., leaves, roots, flowers, etc., are efficient at bioreducing the cations to their metallic counterparts, for instance Au^3+^ and Ag^+^ to yield their respective metallic NPs. Although the biosynthesis of metallic Cu NPs using plants’ extracts resources have been reported, an external source of energy is required, such as heating [49] or microwave irradiation [50], which excludes the case of living plants that grow at room temperature.

In another study, Marshall et al. [51] extended the bioreductive capabilities of B. juncea to promote the production of Au NPs. Regardless of its initial concentration, they found that Au^3+^ was reduced within this plant to ~50% Au(I) and ~50% Au(0), the latter forming NPs of 2–50 nm in diameter. Linear combination X-ray absorption near edge structure (LC-XANES) analysis of the experiments carried out with the living plant, *Sesbania drummondii*, regarding gold distribution within this plant revealed the presence of more than 80% of metallic gold in the roots and shoots, regardless of the initial concentration of KAuCl_4_, the rest consisted mainly of Au_2_S [52]. Transmission electron microscopy (TEM) images showed root cells loaded with Au NPs. Once extracted, these NPs displayed efficient catalytic activity in the reduction of 4-nitrophenol to 4-aminophenol. Feeding another living plant, *Chilopsis linearis*, with different amounts of KAuCl_4_ enabled Rodriguez et al. [53] to determine the threshold of added gold precursor that did not affect the growth of this plant. Moreover, gold accumulation in the plant increased with time. This yielded the in vivo production of Au NPs that could be found in the roots, stems, and leaves. However, the size of the NPs decreased dramatically from their location in the roots to the leaves.

To obtain an insight on the uptake kinetics of silver precursor, Harris and Bali [54] found that the uptake capabilities of *B. juncea* through the roots were not affected by the contact time nor by the AgNO_3_ input while these parameters impacted Medicago sativa, but without any trend. TEM micrographs revealed the presence of nanoparticles of ~50 nm within the plants; however, the chemical state of silver was not reported. To further investigate the internalization of silver precursor, Haverkamp and Marshall [55] fed the same living plant, *B. juncea*, with different silver precursors: AgNO_3_ that provided simple Ag^+^ and 2 different complexes of Ag^+^, Ag(NH_3_)_2_^+^ and Ag(S_2_O_3_)_2_^3−^. As a result, the uptake within the first 8 h was approximately proportional to time, but the uptake was higher when this plant was challenged by AgNO_3_. Their hypothesis is that cationic silver (Ag^+^) is smaller than the 2 other silver complexes, hence its faster uptake. Moreover, in the case of AgNO_3_, silver uptake rate is independent of its initial concentration, denoting a zero-order uptake kinetics under the conditions tested. Plant uptake of different silver precursors yields the production of metallic silver in a proportion that is dependent on both precursor nature and time. Based on their results and other findings from the literature [56], the authors suggested that all redox couples with a standard potential higher than 0.00 V might be subjected to reduction by plants. However, this assumption is too optimistic as there are no reports on the production of metallic copper nanoparticles although the redox couple Cu^2+^/Cu possesses a standard potential of 0.34 V.

**Figure 1:**
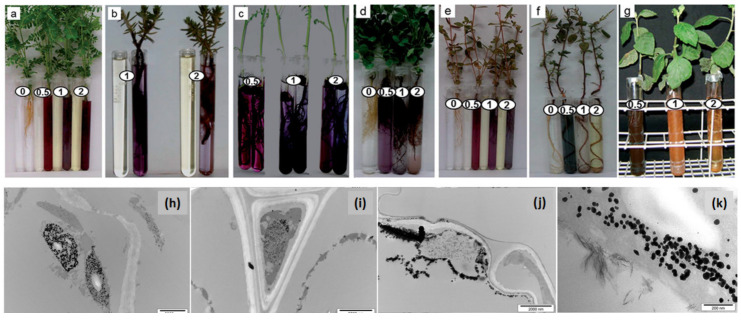
(**a**–**g**) Production of Au NPs by the roots of the live plants: (**a**) *Phyllanthus fraternus*, (**b**) *Portulaca grandiflora*, (**c**) *Cicer arietinum*, (**d**) *Medicago sativa*, (**e**) *Euphorbia hirta*, (**f**) *Amaranthus gracilis* and (**g**) *Vernonia cinerea*. The plants were exposed to 0.0, 0.5, 1.0 and 2.0 mM HAuCl_4_ aqueous solutions. Some controls contain the culture medium supplemented with cationic gold but not the plant. Adapted from Ref. [60] permission from the Royal Society of Chemistry (RSC). (**h**–**k**) Localization of Ag NP within the plant tissues of *Brassica juncea*: (**a**) leaf, (**b**) lower section of outer stem, (**c**) root and (**d**) lower section of inner stem. Adapted from Ref. [61] with permission from the RSC.

Thus far, most of the literature has focused on Ag NPs or Au NPs although Manceau et al. [57] described the formation of metallic copper NPs, but at the soil-root interface of 2 wetland plants, a process assisted by endomycorrhizal fungi. Following a similar procedure, Pardha-Saradhi et al. [58] observed the formation of Fe-nanocomplexes at the root surface of several plants, listed in Figure 1a–g; however, the absence of peaks in the XRD pattern and NP contours and contrast in TEM micrographs clearly showed these NPs lacked crystallinity. A previous study by the same group [59] found that the same plants, listed in Figure 1a–g, that could not synthesize crystalline iron-based NPs [58] promoted the synthesis of both Ag NPs and Ag_2_O NPs at the surface of their roots. This fact, the production of Ag NPs and Ag_2_O NPs, highlights the inability of these plants to fully reduce cationic silver into its metallic analog, as discussed earlier in this paragraph. Since the redox potential of Au^3+^/Au is higher than that of Ag^+^/Ag, the root systems of the same plants were exploited to produce Au NPs ranging from 5–100 nm in size. Figure 1a–g depicts the change in color that occurs in the growth medium of the live plants after the addition of cationic gold demonstrating the production of Au NPs mediated by the roots of the same plants. This change in color is a function of the plant species and cationic gold concentration. Interestingly, all the plants involved in these experiments seem to be in good shape as their leaves have remained green. The as-obtained Au NPs displayed interesting catalytic properties. However, these studies did not determine the yield nor the kinetics of the plant-mediated Au^3+^ bioconversion into Au NPs [60].

In addition to corroborating that the uptake capability of Au^3+^ by the roots is a function of the plant species involved (*vide supra*), the study by Starnes et al. [62] demonstrated that the experimental parameters, namely the reaction time, pH, temperature, and light intensity, could be tuned to target the synthesis inside the roots of Au NPs of desired sizes and shapes. Other studies focused on the sites of metallic NP formation. For instance, Beattie and Haverkamp [61] established that Au NPs and Ag NPs are found in all parts of *B. juncea*, but they are most abundant in the green parts, specifically inside and surrounding the chloroplasts (Figure 1h–k). However, Marchiol et al. [63] reported the opposite; they observed that Ag accumulation in *B. juncea*, whose roots were in contact with an aqueous solution of AgNO_3_, decreased from the roots to leaves. The bioreductive capacities of chloroplasts are clearly demonstrated in several studies discussed in the present review, but how light intensity affects the role played by the green parts of the plants compared to the roots during NP formation has yet to be unraveled as these effects are of great scientific interest. Borovaya et al. demonstrated the synthesis of CdS QDs of 5–7 nm in size using the hairy roots of the living plant *Linaria maroccana* [64] via a 2-step procedure, as described for the production of CdSe QDs using yeast [65]. The living roots were first incubated with CdSO_4_ for 3 days then a solution of Na_2_S was added and allowed to react for another 4 days.

## 3. In Vivo Biosynthesis of Inorganic Nanomaterials Using Living Cells of Microalgae

This section investigates the in vivo production of NPs from algal cell cultures and the factors that affect this process. It is well established that living algae cultures can intracellularly produce nanomaterials as a part of their metabolism [2,66,67,68]. In addition, the cell wall and other cellular biomolecules of both photosynthetically inactive and dead cells have been reported to catalyze the synthesis of nanomaterials [69]. The production, scale-up, and nanomaterial post-processing, i.e., how NPs can be collected, purified, quantified, and stored, will be discussed.

One major advantage of biosynthesized, inorganic NPs is their outstanding colloidal stability, which refers to the ability of inorganic NPs to form homogenous and stable suspensions within their solvent, water in most cases [2]. This allows the biosynthesized NPs to be used directly or post-functionalized with various biological or chemical entities tailored for specific applications. Generally, the colloidal stability is achieved by covalently attaching a polymeric substance or surfactant to the NP surface to prevent aggregation and sedimentation of the NPs [70]. The colloidal stability of algal-synthesized NPs is achieved when natural, high molecular weight biopolymers are secreted by the algae into the culture media and coat the NPs via adsorption. These substances, known as extracellular polymeric substances (EPS) or extracellular matrix (ECM), provide strong steric stabilization to the NPs in the culture media [67,71]. During the in vivo NP biosynthesis, the EPS coating prevents the uncontrolled growth of the NPs and preserves their morphological characteristics.

### 3.1. Effect of Cell Surface on NP Formation

The cell surface chemistry plays a vital role in NP production since the cell surface adsorption-reduction of metal cations contributes more to NP synthesis than intracellular NP formation following the metal uptake [12]. Many studies have reported the cell surface involvement in the production of NPs, as presented in Table 1 [72,73,74,75,76,77]. The negatively charged surface groups of the cells, such as hydroxyl, carboxyl, and phosphate, have an affinity for metal cations [78,79]. The algal cell surface also contains biomolecules with redox capacity, e.g., hydroxyl (-OH), carboxyl (-COOH), amino (-NH_2_), phosphate (-PO_3_), and sulfhydryl (-SH), to reduce metal cations into metallic NPs, causing an NP accumulation on the exterior envelope of the cell [79]. The amount of negatively charged groups on the cell surface depends on the cell culture conditions and the alga’s species-specific surface characteristics [12,80].

In microalgal mediated NP synthesis processes, the cells are generally washed with deionized water (DIW) to give rise to the washed biomass before being exposed to various precursors [5,69,81]. Hereafter, the washed biomass refers to the cells of a given organism that are harvested and washed before being resuspended in deionized water. These washed cells remain viable for long enough to begin growing should they be transferred into a suitable culture medium. The use of washed cells for NP synthesis purposes is a method that separates the cells from the EPS in the cell culture supernatant, but can also remove cell surface biomolecules that could bioreduce precursor ions to metallic NPs. Nevertheless, many studies have reported NP synthesis by this method. For example, Arévalo-Gallegos et al. reported the accumulation of nanosilver on the cell surface of *Botryococcus braunii* that was washed with distilled water before incubation with AgNO_3_ [5]. However, the colloidal stability of the as-produced Ag NPs was not discussed. In this regard, Rahman et al. recently used washed cells of *Chlamydomonas reinhardtii* to produce colloidally unstable Ag NPs whereas both the complete cell culture (i.e., unwashed cells) and cell culture supernatant produced very stable Ag NPs of controlled size and shape [69], confirming that the EPS was required for NP stability. In a similar study, Pytlik et al. reported the formation of Au NPs when the sea water diatom *Stephanopyxis turris* was cultivated with HAuCl_4_. These Au NPs were found to be associated with the diatom cell surface, but the NP stability was not discussed [82].

Although neither of the above studies examined the effects of cell rupture or cell wall integrity after washing or exposure to metal cations, Satapathy et al. reported sustained integrity of *Chlorella vulgaris* cell walls after producing Ag NPs starting from silver nitrate [83]. These Ag NPs aggregated on the cell surface and dislodged during stirring and harvesting, a fact that could facilitate the recovery of these particles.

**Table 1:** List of in vivo synthesized, algae-based nanomaterials.

Nanomaterial	Shape	Size Range (nm)	Precursor(s) or Substrates	Species	Biological Fraction	Synthesis Location	Additional Catalyst(s) or Control Variables	Cultivation Type	Reference
Ag NP	spherical, needle	168–915	photons, AgNO_3_	*Botryococcus braunii*	washed biomass		pH, AgNO_3_ concentration		[5]
Ag NP	rounded, rectangular	5–15/ 5–35	AgNO_3_	*Chlamydomonas reinhardtii*	cell-free extract/ live cell	in vitro/ in vivo	oxidoreductive proteins, reducing capacity of biological extracts, incubation time, nitric acid, HCl	Erlenmeyer flask / agar plate	[6]
Fe-based NP	spherical	0.6–1.0	Fe(II)/Fe(III) ions	*Euglena gracilis*		intracellular		Erlenmeyer flask	[84]
Au NP		<20	Au, HAuCl_4_ xH_2_O	*Lyngbya majuscula, Spirulina subsalsa, Rhizoclinium hieroglyphicum*	washed live algal biomass, live algae	Intracellular	pH, time		[85]
Ag NP	spherical	15–30	photons, AgNO_3_	*Desmodesmus* sp.	living cultures	Intracellular			[86]
Au NP			HAuCl_4_	*Cosmarium impressulum, Klebsormidium flaccidum, Euglena gracilis, Anabaena flos-aquae*	living cells	within cells, cell outer surface, culture medium	20 °C, photons	Erlenmeyer flask	[77]
Au NP	spherical	5.7, 11.3	HAuCl_4_	*Cosmarium impressulum, Kirchneriella lunaris, Euglena gracilis*	living culture	intracellular (thylakoidal membranes)		Erlenmeyer flask	[87]
Ag-Au alloy NP	spherical		photons, AgNO_3_, HAuCl_4_·H_2_O	*Chlamydomonas reinhardtii*	living cells	Intracellular	incubation time	Erlenmeyer flask	[88]
Au NP	spherical	10–10s	HAuCl_4_	*Euglena gracilis*	living cultures	Intracellular		Erlenmeyer flask	[89]
Ag NP	spherical	20, 8.2, 8.8	AgNO_3_	*Spirulina platensis, Chlorella vulgaris, Scenedesmus obliquus*	algae biomass, algal culture	in vivo			[90]
CdS NP		150–175		*Scenedesmus-24*	live cells		pH, initial Cd concentration, biosorbent dose (biomass		[91]
Au NP	spherical, triangular, hexagonal, irregular	3–5 nm, 0–30, 20–50, 100+, 300	HAuCl_4_·3H_2_O	*Anabaena laxa*			Concentration)	Erlenmeyer flask	[13]
Ag NP	spherical	6–24, 15–60	AgNO_3_	*Euglena gracilis, Euglena intermedia*	alga cell suspension, cell-free filtrate	intracellular, extracellular			[73]
magnetic nanomaterial			FeCl_3_·6H_2_O	*Chlorella* sp.	living culture				[92]
Ag NP		53–72	AgNO_3_	*Chaetoceros calcitrans, Chlorella saline, Isochrysis galbana, Tetraselmis gracilis*	algal culture		microwave irradiation, photons		[93]
Au NP	triangular, spherical	25, 30	HAuCl_4_	*Coelastrella* sp., *Phormidium* sp.	algal culture	intracellular			[94]
Au NP	nanorod	137–209 length, 33–69 diam.	HAuCl_4_·xH_2_O	*Nostoc ellipsosporum*	biomass	intracellular			[95]
Au NP	spherical, irregular, nanorods	8–42, 10–30	HAuCl_4_·xH_2_O	*Leptolyngbya tenuis, Coleofasciculus chthonoplastes, Nostoc ellipsosporum*	biomass	extracellular		agar slab, flasks, tub/tank culture	[96]
Au NP	spherical, triangular	10–30	HAuCl_4_	*Stephanopyxis turris,*	living culture				[82]
Au-silica nanocomposite				*Stephanopyxis turris,*	living culture				[97]
Ag NP	spherical	~ 3–8	photons, AgNO_3_	*Chlamydomonas reinhardtii* (wild type strain)	living culture			Erlenmeyer flask	[98]
Ag NP	spherical	~3–8	photons, AgNO_3_	*Chlamydomonas reinhardtii* (cell wall deficient strain)	living culture			Erlenmeyer flask	[98]
Ag NP	spherical	~2–24	photons, AgNO_3_	*Chlamydomonas reinhardtii*	living culture		quantum efficiency of the cells	Erlenmeyer flask	[69]
Ag NP	irregular	n/a	photons, AgNO_3_	*Chlamydomonas reinhardtii*	washed cells		quantum efficiency of the cells	Erlenmeyer flask	[69]
Cu NP		15–65	CuSO_4_·5H_2_O	*Chlorella kessleri, Dunaliella tertiolecta, Tetraselmis suecica*	living culture				[99]
Ag NP		8–20, 12.62	AgNO_3_	*Chlorella vulgaris*				continuously stirred non-aerated culture assembly	[83]
Au NP	spherical, triangular	5–35	HAuCl_4_	*Tetraselmis kochinensis*	harvested cells			algal culture chamber	[100]
Pd@Ag core-shell NP	spherical	5.37–38.98		*Spirulina platensis*	washed cells				[81]
Ag NP	spherical	<200	AgNO_3_	*Phaeodactylum tricornutum*	living culture			Erlenmeyer flask	[101]
biosilica				*Nitzschia closterium, Thalassiosira*	dry biomass				[102]
CdSe QD	spherical	5–6	Na_2_SeO_3_, Cd(NO_3_)_2_	*Chlorella pyrenoidosa, Scenedesmus obliquus*		intracellular	dosage, pH, reaction temperature, and time	Erlenmeyer flask	[103]
CdSe NP	spherical		Na_2_SeO_3_, Cd(NO_3_)_2_	*Selenastrum capricornutum, Microcystis aeruginosa*		intracellular			[104]

Algal flagella can also promote the synthesis of nanoparticles, as reported by Barwal et al. [6]. In addition to the internalization of Ag NPs by the flagella, this group reported a distribution of particles outside the flagella. However, more flagella-located Ag NPs were internal than external, as indicated by the distention of the flagella. NP formation on the cell surface or outside of the flagella may be significantly less than the extracellular formation by EPS or intracellular formation by the cell. For instance, Rahman et al. used the wild type and the cell wall deficient strains of *C. reinhardtii*, but did not observe any differences in Ag^+^ to Ag NP conversion in the presence or absence of the cell wall. Therefore, cell wall components did not significantly enhance nor inhibit Ag NP formation and the reaction was likely controlled by the EPS-facilitated reduction of Ag^+^ [98]. Nevertheless, further experiments are needed to completely understand the contribution of the cell surface and its role in the production of nanomaterials. Additionally, the stability and recovery of the as-produced nanomaterials will be further discussed in the current review.

### 3.2. Factors Affecting the In Vivo Synthesis Process

Several factors including the temperature, pH of the reaction medium, incubation time, illumination intensity, precursor ion concentration and metal cation to biomass ratio can affect the production and morphology of in vivo synthesized nanomaterials [2,7]. However, it is worth noting that the influence of these factors can vary depending on the strain of algae used, owing to the differences in their enzymes and/or cell wall constituents [13,77]. Table 2 shows a range of nanomaterial synthesis parameters using different microalgal species.

The extracellular production of nanomaterials using algae (such as algal dry powders, liquid extracts, etc.) have mostly been performed at temperatures between 20 °C and 30 °C (Table 2), but synthesis temperatures as high as 100 °C have been reported [105,106]. For example, Lengke et al. reported the intracellular formation of <10 nm Ag NPs by the filamentous cyanobacterium, *Plectonema boryanum*, at temperatures ranging from 25 °C to 100 °C [107]. The same study reported extracellularly produced Ag NPs with sizes from 1–200 nm. This result indicates that a tight control over the size of the Ag NPs is achieved when produced in vivo compared to the extracellular pathway at temperatures up to 100 °C. However, a further study at different temperatures is needed to attribute the impact of the temperature on the morphology of the as-produced nanomaterials and shed light on the nature and state of the biomolecules involved in this process.

The precursor concentration often determines whether the formation can take place intracellularly or extracellularly. At higher metal precursor concentrations, the cells fail to maintain their integrity, burst, and release the cell components into the surrounding environment, as previously reported [5,108]. In this context, the extracellular synthesis can be a better alternative as it allows for higher precursor concentrations to be used, which could also accelerate the reaction [5,109]. Although *E. gracilis* continued to grow in the presence of iron ions, neither the biomass nor the precursor concentrations were determined in the study by Brayner et al. [84]. In another study, Au NPs produced using *Rhizoclonium hieroglyphicum* and *Lyngbya majuscula* indicated that the cells were viable throughout the experiment at a precursor to biomass ratio of 0.06 mM g^−1^ [85]. According to Table 2, a maximum of 2 mM g^−1^ precursor to biomass ratio has been explored; however, the study did not report the cell viability of *Coelastrella* sp. or *Phormidium* sp. The precursor concentration to biomass ratio can also impact the shape of the NPs, particularly when one of them becomes the limiting reagent [5]. For instance, Arévalo-Gallegos et al. found that needlelike Ag NPs were produced only at 5 mM of AgNO_3_, as opposed to spherical NPs at lower concentrations, while exploring the intracellular synthesis with concentrations ranging from 1–5 mM. In a recent study, Lenartowicz et al. used *Anabaena laxa,* a freshwater strain of cyanobacteria, to produce Au NPs and reported a correlation between the initial Au^3+^ concentration (0.1–1.0 mM) and the formation of Au NPs of various sizes and shapes [13]. At the highest concentration of Au^3+^ (1.0 mM), *A. laxa* produced triangular, hexagonal and irregular forms of Au NPs that ranged from 20 nm to greater than 100 nm in size. These non-spherical shapes of Au NPs agreed with the needlelike Ag NPs produced at higher AgNO_3_ concentrations, as reported above [5]. In contrast, at 0.1 mM, *A. laxa* produced small and spherical Au NPs with approximate diameters of 3–5 nm. The group attributed the difference to the fact that the Au NPs lacked colloidal stability at higher Au^3+^ concentrations and further correlated this fact with the tendency of these Au NPs to accumulate in the proximity of the cell wall, mainly on the cell surface biomolecules. These results indicate that the quantification of EPS is necessary to predict the colloidal stability and to direct the size and shape of the in vivo synthesized NPs.

It is well known that the pH controls the adsorption of metal ions by protonation and deprotonation of metal binding sites [91]. However, the ion adsorption process may vary between algal species due to differences in the cell surface composition. For example, Chakraborty et al. used several pro- and eu-karyotic algal genera to study the adsorption kinetics of Au^3+^ at pH values of 6, 7 and 8 [85]. The group reported that the biosorption process was almost independent of pH for *L. majuscula*, though initial adsorption was much higher at pH 8. In contrast, *Spirulina subsalsa* showed faster adsorption at pH 6–7 compared to pH 8. More interestingly, when these two species were compared, the adsorption of Au^3+^ by *L. majuscula* (~96.4%) was always higher than that of *S. subsalsa* (~86.4%) at any given pH. Parial et al. conducted similar experiments using three cyanobacterial strains, including *Leptolyngbya tenuis*, *Coleofasciculus chthonoplastes* and *Nostoc ellipsosporum* at different pH values (pH 5, 7 and 9) to monitor the effect of the pH on the Au NP production [96]. The group reported that acidic pH (pH 5) promoted Au NP synthesis, which gradually decreased as the pH increased. In contrast, Arévalo-Gallegos et al. obtained needlelike Ag NPs at a neutral pH, while exploring the intracellular synthesis at several pH values between 6 and 9. Moreover, the group reported NPs with more uniform size and shape under basic conditions when compared to acidic ones [5]. Previous studies reported a higher yield of Ag NPs and faster reduction of Ag^+^ at basic pH levels [109]. For the current discussion, we conclude that the pH can play an important role in the reaction mechanism of NP biosynthesis by determining the (i) adsorption rate of precursor ions, (ii) NP yield and (iii) morphology of the NPs.

### 3.3. Post-Processing of In Vivo Synthesized Nanomaterials

To date, no systematic review of the post-processing of algal-synthesized nanomaterials has been published. The major post-processing steps include the collection or recovery, purification, quantification, and storage of the as-biosynthesized NPs. It is worth noting that these post-processing steps can be applied to any in vivo or in vitro microbial NP synthesis process. However, two important factors to consider in the post-processing are the colloidal stability of the NPs and the location of their synthesis since these factors determine the process. Internally synthesized NPs have been reported to diffuse out of cells (e.g., *C. reinhardtii*) with an EPS coating, which provides colloidal stability to the NPs. The EPS-coated NPs are reported to be colloidally stable from several hours to a few months in various solutions, including saline water, acetone and ethanol [88,98]. Although various methods, such as sedimentation [87], filtration [83] and centrifugation [110], have been proposed and used to collect intracellular NPs, no real attempts to quantify the NP amount have been carried out [11].

The ultrasonication is one of the most widely used techniques to disrupt the cells to recover the intracellular NPs [111]. When the NPs are strongly bonded to intracellular components, robust recovery methods are needed. One possible method is the wet heat sterilization process that was used to recover selenium NPs from bacterial cells [110]. The method includes passivation of the cells at 121 °C and 118 kPa gauge pressure for 20 min to disrupt the cells and release the NPs that were then treated by an “extracellular recovery” method (i.e., centrifugation) before being purified by washing and sonication. This method is advantageous because it does not chemically change the NPs. Although no disadvantages were reported, there could be irreversible thermal denaturation of protein fraction of the EPS that are often found to be associated with the colloidal stability of algal-synthesized NPs [112]. However, a recent study reported thermal denaturation temperatures of up to 107 °C for protein concentrates and isolates developed from the algal species *Spirulina* sp. LEB 18. This heat sterilization process as a cell disruption technique in algal NP downstream processing is a possible area for future research [113].

To recover the surface-bound NPs, Satapathy et al. used ammonia to harvest almost all the Ag NPs that were adsorbed on the external surface of the cell wall of *C. vulgaris* [83]. The NPs were then separated from the ammonia solution by filtration and the purified Ag NPs were recovered by evaporating the remaining ammonia solution. This method led to more desorption of Ag NPs from the cell surface than by high speed centrifugation. However, the Ag NP recovery was not quantified therefore the effectiveness of this method requires further investigation.

The purification of algal-synthesized NPs is an important step to remove unreacted precursors and contaminants and increase the NP quality. The purification can be done through physical or chemical methods. The physical methods include centrifugation [83,110], sonication [110,114], sedimentation [115] and washing with distilled water [110,115] to remove any biological matter, free ions, and residual media components. Although these are all commonly used methods to purify NPs, they do have their drawbacks. Sedimentation under gravity is inexpensive, but it can be time consuming. It was reported that 1 h of sedimentation under gravity was required to separate Au NPs from the biomass [115]. The same group centrifuged the biomass to extract the Au NPs, but noted that the installation of a centrifuge may increase the production cost. Moreover, Rahman et al. reported that the sedimentation never occurred for long-term, very stable Ag NPs [98]. Therefore, the feasibility of a method often requires multiple factors to be considered, such as (i) the stability of the NPs, (ii) the cost of purification and (iii) time constraints. In this context, a combination of multiple methods is often used to completely purify the NPs.

Chemical treatments using thiourea [85], ammonia [83], dilute hydrochloric acid [85,97], concentrated nitric acid [85,97] and chelators, such as EDTA [85,97], have been effective to desorb Ag NPs from the biomass. Chakraborty et al. found the thiourea to be the best washing reagent as it recovered the Au NPs with 83–100% efficiency from three different algal species [85].

**Figure 2:**
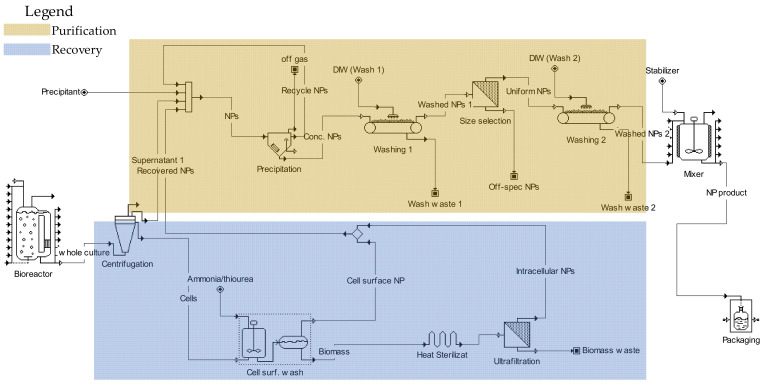
Post-processing flowchart of in vivo synthesized nanomaterials.

Figure 2 shows a PFD that generalizes the downstream processing of in vivo synthesized nanomaterials. The process consists of NP recovery and purification units, as depicted by the color legends in the figure. The process begins with a bioreactor that produces nanomaterials using living cultures of algae or other microorganisms. The centrifugation is used to separate the cell culture into biomass and supernatant streams. The biomass stream from the centrifuge goes into the recovery process while the supernatant goes directly to purification.

In the first step of the NP recovery process, the biomass stream from the centrifuge is washed with thiourea to remove the NPs that are adsorbed on the external cell surface [83,85]. In the next recovery steps, wet heat sterilization and ultrasonication disrupt the cells to recover the intracellular nanomaterials [110,111]. Both the surface and intracellular nanomaterials streams from the recovery process feed the purification unit.

The cell culture supernatant from the centrifugation and the NP streams from the recovery process comprise the feed to the purification process. Alternatively, the three streams could be purified separately if their properties are significantly different. The purification process starts with the precipitation of the nanomaterials, for example, using ethanol or acetone, followed by the successive washing with deionized water [110,115]. The size selection is then performed to eliminate off-spec NPs. In the final step of post-processing, nanomaterials are stabilized by providing stabilizers, such as starch, from an external source. The stabilization step is needed if the processed NPs lack enough EPS for the colloidal stabilization, such as when the EPS is removed in the case of washed cells, as discussed in Section 3.1.

Only a few studies have quantified the production of biosynthesized NPs. This quantification can be done directly by measuring Ag in Ag NPs or indirectly by measuring the unconverted free ions by inductively coupled plasma (ICP), atomic emission spectroscopy (ICP-AES), mass spectrometry (ICP-MS) or with an ion-selective electrode (ISE). For instance, one method to directly quantify Ag NPs using ICP-AES has been reported by Rahman et al. [98]. The Ag in Ag NPs was determined after removing the unreacted Ag^+^ by complexing it with Cl^-^ yielding the precipitation of AgCl owing to the addition of concentrated NaCl solution. The conversion was then determined by comparing the Ag in Ag NPs to the total Ag as AgNO_3_ input to the reaction. Therefore, the method required an effective separation of free ions from the NPs. This method was effective because the Ag NPs were highly stable in the NaCl solution. In another study, the conversion of Ag^+^ to Ag NPs was assessed by quantifying the Ag in the supernatant by ICP-MS, after the Ag NPs were removed by centrifugation [6]. However, the mass of Ag NPs was not determined by any of these groups nor were the intracellular and cell surface-produced NPs differentiated. This quantification could be done by proper collection and purification methods (*vide supra*) and then determining the NP quantity contributed by individual biological fractions.

Biosynthesized NPs tend to agglomerate or sediment during long-term storage, leading to a loss of nano-inherent properties, which diminishes their potential applications [33]. Ag NPs biosynthesized by *C. reinhardtii* and stabilized by EPS have been reported to remain stable for more than 300 days when stored at 4 °C in the dark [98], thus proper stabilization techniques could enable long-term storage without deleterious effects. Additional stabilizers can also be used to further stabilize the nanomaterials, as discussed in Figure 2.

## 4. In Vivo Biosynthesis of Inorganic Nanomaterials Using Yeasts

The in vivo synthesis of nanomaterials using yeast began in 1989 when Dameron et al. reported the intracellular production of cadmium sulfide QDs of ~2 nm in size after exposing *Candida glabrata* and *Schizosaccharomyces pombe* yeast cultures to cadmium precursors [116]. The yeast adapted to the presence of the Cd salts in the growth medium by producing short peptides that chelated and controlled the growth of the CdS nanocrystals. Using the same species, Williams et al. [117] used a culture freeze-thaw method to release the intracellularly yeast-produced CdS NPs without breaking the cells. The same authors investigated the optimal fed-batch conditions to synthesize CdS crystals using *S. pombe*. They found that adding cadmium sulfate during the mid-exponential growth phase enabled the concomitant uptake of Cd precursor and inorganic sulfide yielding the formation of CdS crystals [118]. In another paper, they also determined the optimum conditions capable of maximizing *S. pombe* biomass yield for an enhanced production of CdS QDs [119]. A similar investigation was carried out by Krumov et al. [120] using *S. pombe* and *C. glabrata* where they compared Cd uptake by both species and demonstrated that CdS formation was exclusively intracellular as the NPs were evenly distributed within the cell cytoplasm. Moreover, phytochelatins were determined to be the capping agent of the as-produced QDs. Additionally, washed cells of *S. pombe* were reported to promote the intracellular production of ZnS QDs of 30–40 nm in diameter [121].

**Figure 3:**
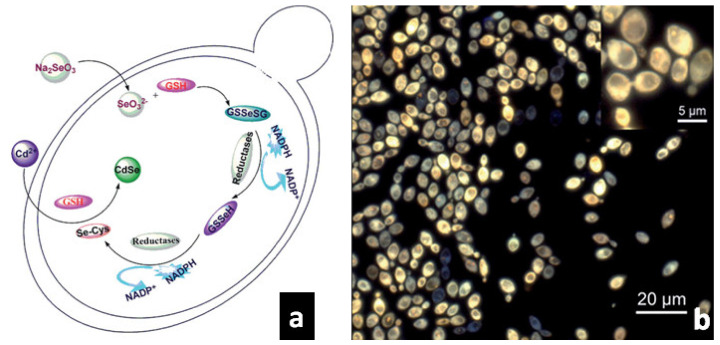
(**a**) Scheme of 2-step procedure for the synthesis of CdSe QDs: first, the added Na_2_SeO_3_ precursor enters the cells of *Saccharomyces cerevisiae* where Se reacts with GSH to give rise to organoselenium compounds; then CdCl_2_ is added, enters the cells and reacts with organoselenium compounds to yield CdSe QDs by reacting. (**b**) Fluorescence images of *S. cerevisiae* cells that produce intracellular CdSe QDs via the 2-step procedure described in (**a**). Adapted from Ref. [65] with permission from Wiley.

*Saccharomyces cerevisiae* can efficiently biosynthesize CdSe and CdTe QDs. For instance, Cui et al. [65] produced CdSe QDs via a 2-step procedure (Figure 3a). The live stationary phase yeast cells were co-incubated with Na_2_SeO_3_ for 24 h then CdCl_2_ was added to this mixture and shaken in dark for 10–40 h. Owing to the action of glutathione (GSH), an antioxidant found in most aerobic organisms [122], Se^4+^ is reduced into organoselenium compounds during the first step, resulting in the production of CdSe QDs during the second step. Fluorescence microscopy shows that the produced intracellular QDs are less than 3 nm in diameter (Figure 3b). Moreover, the concentration of the precursors impacts the biosynthetic process. Above a certain concentration, the metabolism of the cells is inhibited, thus hindering the synthesis of CdSe QDs. The same group also performed the synthesis procedures using both the wild type (WT) *S. cerevisiae* and a GSH-deficient mutant of the same species to determine the role of GSH in the biosynthesis of CdSe QDs [123]. As a result, the GSH deficient mutant displayed a lower intracellular CdSe QD production when compared to the WT strain. On the other hand, strains with galactose-inducible GSH genes produced more CdSe QDs than the WT. These findings highlight the significant role played by GSH in the *S. cerevisiae*-mediated synthesis of CdSe QDs.

In addition to corroborating the findings of Cui et al. regarding the use of yeast cells at their stationary phase of growth [65], Wu et al. investigated the optimization of CdSe QD synthesis using live cells of *S. cerevisiae* by screening the impact of the time of addition, the amounts and incubation time of Na_2_SeO_3_ and CdCl_2_ with Na_2_SeO_3_ [124]. In another study, Bao et al. used the same species of yeast to carry out the fabrication of well-dispersed CdTe QDs of ~3.6 nm in diameter [8]. However, the process occurred in the presence of mercaptosuccinic acid and sodium borohydride.

Live cells of different species of yeast have been exploited to produce metallic NPs. For instance, Kowshik et al. synthesized Ag NPs by adding AgNO_3_ to the mid-exponential growth phase strain of MKY3 [125]. Elahian et al. used a *Pichia pastoris* mutant that overexpresses a metal-resistant variant of cytochrome b15 reductase enzyme to promote the production of Ag NPs and Se NPs [126]; the same mutant was also screened for its ability to produce Au NPs and Pd NPs [127]. Lin et al. demonstrated that the dry biomass of *P. pastoris* WT strain could act as an adsorbent for AuCl_4_^-^ and, subsequently, promote the formation of Au NPs that display interesting catalytic activities [128]. The chemical modification of the cell surface of the same species does not seem to bring any significant advantage regarding AuCl_4_^−^ adsorption when compared to its WT parent [129].

Agnihotri et al. used washed cells of *Yarrowia lipolityica* to biosynthesize Au NPs [130]. As a follow-up, the same group studied the impact of *Y. lipolytica* biomass amount and gold precursor concentration on the morphology of the Au NPs synthesized either by the live cells or their supernatant [131]. Scanning electron microscopy (SEM) micrographs show Au NPs of different shapes and sizes on the surface of *Y. lipolytica*. Fluorescence imaging of the cells would aid in accurately determining the formation site of these Au NPs, as previously demonstrated in the study of CdSe QD synthesis using yeast (*vide supra*). TEM is also an excellent technique to study the size and the shape of the NPs, either synthesized in the supernatant or released to it [132]. For example, TEM imaging of washed cells of *Pichia jadinii* [133] and *S. cerevisiae* [134] show Au NPs inside the cells. In addition to metallic NPs, autoclaved, deactivated biomass of *S. cerevisiae* keeps its reductive capabilities while still promoting the formation of metalloid Se NPs [135].

## 5. In Vivo Biosynthesis of Inorganic Nanomaterials Using Fungi

Muraly Sastry and collaborators carried out pioneering work in the biosynthesis of nanomaterials. Working extensively with the washed biomass of the fungus *Fusarium oxysporum* in a distilled water-cell suspension at room temperature and atmospheric pressure, they extracellularly produced NPs made of gold [136], silver [137], bimetallic alloy of gold and silver [138], cadmium sulfide [139], zirconia [140], silica and titania [141], and magnetite [142]. Interestingly, the composition of the bimetallic Au-Ag alloy NPs they obtained is controlled by the amount of *F. oxysporum* washed biomass used rather than by the ratio of the introduced salts (50:50 Au:Ag); the higher the biomass concentrations, the higher the Ag:Au molar ratio in the NPs. However, the authors did not discuss the fate of the unreacted salts [138]. On the other hand, the washed biomass of the fungus *Verticillium* sp. enabled exclusively the intracellular production of Ag NPs [143] and Au NPs [144], but the addition of an aqueous solution of 2:1 molar of K_3_[Fe(CN)_6_] and K_4_[Fe(CN)_6_] triggered the extracellular synthesis of mainly magnetite NPs [142]. Analyses carried out on these Fe_3_O_4_ NPs allowed the isolation of a 55 kDA protein from the fungal extract that was able to induce the production of the magnetite NPs once in contact with the iron precursors. Furthermore, 21 kDa and 24 kDA proteins isolated from the fungus *F. oxysporum* are believed to be responsible for the extracellular biosynthesis of silica [141] while slightly heavier proteins, i.e., 24 kDa and 28 kDa, from the same fungus are shown to be involved in the extracellular biosynthesis of zirconia [140]. Using the alkalotolerant fungus, *Trichothecium* sp., the same group directed the biosynthesis of Au NPs to be extracellular or intracellular by carrying out the reaction under stationary or shaking conditions, respectively [145]. The former gave rise to spherical, rod-like and triangular Au NPs of different sizes while the latter led to the formation of mainly spherical Au NPs. It would be of great interest to determine the yield for each route.

Gericke et al. studied the impact of reaction sets on the fungal production of Au NPs by varying the pH, reaction temperature, cationic gold concentration and growth phase of the cells [133]. For instance, the washed biomass of *Verticillium luteoalbum* enabled the production of Au NPs at all tested values of pH (3, 5, 7 and 9); however, the size and the shape of these NPs varied dramatically with varying pH. Although it does not have an impact on the size of the as-produced Au NPs, the growth phase seems to impact the ability of the cells to take up the gold cations and promote the intracellular synthesis. On the hand, higher concentrations of cationic gold produced aggregated, polymorphic Au NPs while higher temperatures offered better accumulation kinetics of Au^3+^ within the cells. In a similar study, Mishra et al. synthesized Au NPs using the biomass, the cell-free filtrate and the dead cell filtrate of *Penicillium brevicompactum*; however, only the pH and cationic gold input were varied [146]. The extracellularly fabricated Au NPs were successfully tested for their biological activity against mouse cancer cells. The filamentous fungus *Neurospora crassa* triggered the intracellular production of Ag NPs, Au NPs and the bimetallic Ag-Au alloy NPs [147]. In addition to obtaining pure samples of Ag NPs and Au NPs, this team challenged the biomass of *N. crassa* with different molar ratios of Ag^+^ and Au^3+^ to produce bimetallic Ag-Au alloy NPs. Although TEM-EDS analysis confirmed the formation of bimetallic Ag-Au alloy NPs of the sample made of 50:50 Ag:Au, the absence of quantifying methods and UV-Vis spectra does not allow correlation of the Ag:Au molar ratio in the as-produced bimetallic Ag-Au alloy NPs by *N. crassa* to the initial Ag:Au molar ratio input.

As a result of heavy cation detoxification using fungal bioreactors, selenium complexes may be reduced to metallic selenium as indicated by the red-orange color of the fungal pellets [148] or Se NPs as portrayed by field emission transmission electron microscopy (FETEM) images on the fungus cells [149]. To carry out the biosynthesis of Se NPs and Te NPs, Liang et al. [150] cultured four species of fungus, *Aureobasidium pullulans, Mortierella humilis*, *Trichoderma harzianum* and *Phoma glomerata*, in a suitable medium amended with either 1 mM sodium selenite (Na_2_SeO_3_) or 1 mM sodium tellurite (Na_2_TeO_3_), as salt precursors of Se and Te, respectively, while sodium selenate (Na_2_SeO_4_) was discarded owing to its toxicity. In addition to a diverse response in terms of pH changes and biomass yields, the uptake of the salts by the fungi depends on the species, nature of the salt and contact time. For instance, *A. pullulans* takes up less than 50% selenite and more than 90% of tellurite during the study period (30 days). On the other hand, the uptake of selenite and tellurite by *P. glomerata* is time-dependent as it increases over time while tellurite gives a better uptake yield by the same species. This process gives rise to the growth of NPs on the cell surface and in the supernatant of the 4 fungal species used; the features of the as-obtained NPs, mostly Se NPs and Te NPs, are species-, salt-, and site of growth-dependent. Calculations based on salt uptake reveal that for all species, higher NP synthesis yields are obtained with tellurite. Although *A. pullulans* exhibits the highest accumulation of sodium tellurite in its cells, it also displays one of the lowest yields among all species in terms of Te^4+^ to Te^0^ conversion; on the other hand, *P. glomerata* occupies the extreme positions in terms of NP synthesis yield: the lowest for Se NPs and the highest for Te NPs.

The extracellular biosynthesis of various QDs using different species of fungus has been reported by several teams. For instance, *F. oxysporum* was used to produce ZnS QDs [151], CdTe QDs [152] and CdSe QDs [153,154]. On the other hand, the biomass of *Phanerochaete chrysosporium* [155] and *Trametes versicolor* [156] produces CdS QDs, *Aspergilus flavus* [157] synthesizes ZnS QDs and *Helminthosporum solani* yields CdSe QDs [158]. Remarkably, the biosynthesis of selenium- and tellurium-based QDs using these fungal species are all carried out via a single-step process by mixing the washed biomass of the fungi with the corresponding precursors of the elements making the QDs. On the other hand, the production of CdTe and CdSe QDs using yeast was performed via a 2-step process where the Se or Te precursor is first added and let to incubate with the yeast cells for several hours prior to the addition of Cd cations (*vide supra*).

## 6. In Vivo Biosynthesis of Inorganic Nanomaterials Using Mammalian Cells and Mammals

This route remains the least explored among the aforementioned methodologies for several reasons: (i) the difficulty of culturing mammalian cells (with the right cell line, regulatory issues, required lab equipment, time and cost), and (ii) the fear of transfection-infection-contamination issues should the as-synthesized NPs be used in any application related to the biomedical field. Thus far, only a handful papers relating the biosynthesis of Au NPs, Ag NPs, and Fe_3_O_4_ NPs using mammalian resources have been published.

In 2005, Anshup et al. [159] were the first to ever report on the synthesis of Au NPs of 20–100 nm in diameter after adding 1 mM aqueous tetrachloroauric acid (HAuCl_4_) to several lines of mammalian cells including non-malignant HEK-293 (human embryonic kidney), malignant HeLa (human cervical cancer), SiHa (human cervical cancer) and SKNSH (human neuroblastoma) cell lines and followed the Au NP production kinetics using ultraviolet-visible (UV-Vis) spectroscopy. For all the cell lines, UV-Vis revealed a progressive release of Au NPs, but not all the synthesized Au NPs were released to the supernatant as the SPR band of lysed cells after 96 h incubation with cationic gold was more intense than that of the supernatant recorded on the same date. Furthermore, non-malignant cells released higher amounts of Au NPs to the supernatant. The intracellular character of this process was further corroborated using optical microscopy and TEM. In another study, Jin et al. [160] reported the intracellular synthesis of Au NPs when HAuCl_4_ was added to platelet suspensions in the presence of sodium citrate and sodium borohydride. In one experiment, this mixture was subjected to ultrasound irradiation whereas the other one was not. Although the authors recovered Au NPs synthesized within the platelets, they did not present any quantification regarding the molar ratio of cationic gold that was reduced intracellularly vs. via an extracellular process. Moreover, the presence of sodium citrate and sodium borohydride, widely used to produce Au NPs [161] does not help in determining the accurate role played by the platelets in this process. Furthermore, although the authors claim that the use of ultrasounds improved the yield of Au NPs synthesis, this might not be the case based on their data. In fact, UV-Vis spectra on lysed cells exhibit SPR bands of almost the same intensity for both samples; this is corroborated by the same amount of Au NPs per cell obtained using atomic absorption spectrometry. It would be interesting to carry out the same experiment without adding sodium citrate and sodium borohydride or using ultrasounds; this will allow for the assessment of the ability of the platelets to carry out the intracellular synthesis of Au NPs and determine the yield. The Au NP fabrication and presence did not hinder the platelets from aggregating after the addition of thrombin, indicating that these Au-loaded blood cells maintained their biological activity.

**Figure 4:**
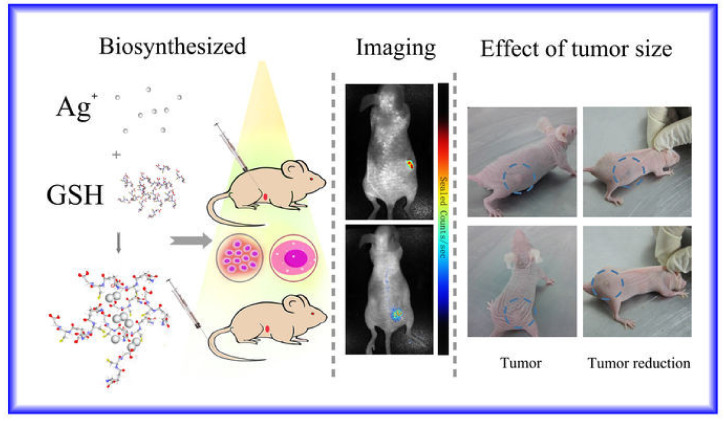
Schematic of administration of [Ag(GSH)]^+^ complex solution to mice bearing tumor xenograft, followed by the subsequent formation of Ag NCs that are exploited in both NIR-fluorescence imaging and treatment of cancer. Reprinted from Ref. [165] under creative common license agreement.

These few studies focus on the exploitation of mammalian cells to promote the production of Au NPs. A plausible explanation for the choice of this noble metal may lay in its high standard potential– E°(Au^3+^/Au) = 1.52 V. This makes cationic gold easy to reduce into its metallic counterpart by the diverse biomolecules that are present in the cells, on their surface or released to their supernatant, such as sugars and proteins, to form Au NPs. In fact, several papers have described the synthesis of Au NPs using these biomolecules in aqueous solutions at room temperature [162,163,164]. Following a modified procedure, Gao et al. demonstrated that the synthesis of Ag NPs was more efficient when a solution of silver nitrate that was previously mixed with glutathione (GSH) was added to 3 human cancer cell lines, HepG2 cells, HeLa cells, and the lung cancer cells A549 [165]. Indeed, the viability of the cells significantly increased when AgNO_3_, whose standard potential equals 0.80 V, had been previously mixed with GSH to give rise to the [Ag(GSH)]^+^ complex. This is expected because GHS is known to be a major cellular antioxidant found in most aerobic organisms [122]. As a follow-up to this first experiment, the same group administered 10 mM of [Ag(GSH)]^+^ complex solution via either the tail vein or directly to the tumor in mice bearing xenograft tumors [165]. Regardless of the injection route, the [Ag(GSH)]^+^ complex solution promoted the appearance of silver nanoclusters (Ag NCs) within the tumor while such synthesis did not occur in non-cancerous tissues. Taking advantage of the near-infrared fluorescence of these Ag NCs, it was possible to image the tumor within a few hours post-injection (Figure 4). Ex vivo analyses did not reveal any presence of Ag NCs in any other organs of the body. Moreover, the mice treated with the [Ag(GSH)]^+^ complex solution saw their tumors gradually receding before totally disappearing and their health improving. Another study described the synthesis of ~5-nm Ag_2_S QDs with an emission band at 945 nm by growing the HepG2 cancer cells in the presence of AgNO_3_ and Na_2_S [166]. Interestingly, these QDs are capped by intracellularly produced GSH and were tested successfully in fluorescence imaging in living mice in the near-infrared (NIR) region.

An innovative and emerging methodology to exploit mammalian cells in nanotechnology consists of inserting genes coding for magnetosomes, i.e., magnetite (Fe_3_O_4_) crystals surrounded by a lipidic bilayer, taken from the magnetotactic bacteria (MTB) into the genome of these cells, via transfection, to intracellularly produce magnetic magnetite nanocrystals that are retained within the cells. Zurkiya et al. [167] inserted the *magA* gene, putatively involved in iron transport in MTB, into the human cell line 293FT and controlled its expression by the antibiotic doxycycline. In addition to XRD that displayed peaks matching the pattern of magnetite, TEM on the successfully transfected cells, named hereafter 2B5, showed intracellular vesicles containing varying number of magnetite NPs, after they had been fed with doxycycline and an iron precursor. Theses biogenic NPs behaved as a contrast agent for magnetic resonance imaging (MRI). No toxicity due these NPs was found. A few other studies carried out either on mouse neuroblastoma (N2A) cell line [168] or on human MDA-MB-435 breast/melanoma cells [169] confirmed the possibility of magnetite intracellular biomineralization that relies on transfection using the MTB *magA* gene yielding the production of efficient MRI contrast agents. Although Pereira et al. challenged [170] these findings from several points-of-view, such as the toxicity, the iron uptake, and MRI imaging, Liu et. al. [171] extended the efficacy of MTB *magA* gene when transfected into the genome of mouse multipotent P19 cells to promote the intracellular production of magnetite NPs as an efficient MRI contrast agent.

Instead of relying on the *magA* gene, Elfick et al. opted for the transfection of human mesenchymal stem cells with *mms6* gene, one of the genes coding the production of magnetite nanocrystals in MTB [172]. In addition to promoting the intracellular production of magnetite NPs, this procedure enabled the cells to keep their viability while retaining the NPs. Importantly, the presence of magnetite NPs within the transfected cells did not show any adverse effects on their capacity of proliferation, migration, and differentiation when compared to the WT.

## 7. Conclusions

The present review extensively describes the significance of the in vivo biosynthesis of inorganic nanomaterials using live eukaryotic cells and organisms. Unlike biomineralization, the production of nanomaterials described here is not a result of the metabolism of these biological organisms and occurs solely when the corresponding precursors of the desired nanomaterials are made available to them after which their enzymatic machinery and biomolecules are involved in this process. Via the in vivo biosynthesis routes, materials scientists have succeeded in producing nanoparticles composed of metals, metalloids, oxides, and chalcogenides, using eukaryotic cells under environmentally friendly conditions. Here, we have critically reviewed the nanoparticle synthesis methodologies and reaction mechanisms, and compiled a post-processing scheme for these nanomaterials. Additionally, we have examined the advantages of such processes and pointed out their limitations. In vivo produced nanoparticles exhibit novel and interesting properties and are used in several important applications, such as catalysis, bioimaging, and cancer research and therapy. Although the methodologies described in this review enabled the fabrication of unique nanomaterials either via a suitable experiment design (e.g., CdSe QDs) or as a result of the introduction of relevant genes via genetic engineering techniques (e.g., Fe_3_O_4_ NPs), there is much in the way to discover and investigate to address the many significant challenges to using these nanomaterials, such as cytotoxicity and safety issues, and meeting the key criteria for the design of bioreactors for the green, scalable in vivo production of these nanomaterials through fully and tightly controlled procedures that are clearly important to the booming fields of nanoscience and nanotechnology.

## Figures and Tables

**Table 2:** Factors affecting the in vivo synthesis process.

*Species*	Produced Nanomaterial	Shape	Size (nm)	Temp. (°C)	pH	Incubation Time	Illumination (μmol m^−2^ s^−1^)	Prec. Conc. (mM)	Biomass Weight (g)	Prec. Conc./Biomass (mM g^−1^ Biomass)	Ref.
*Botryococcus braunii*	Ag NP	spherical, needle	168–915	20	6–9	2 d	300	1–5	5 (wet)	0.2–1	[5]
*Lyngbya majuscula, Spirulina subsalsa, Rhizoclinium hieroglyphicum*	Au NP		<20		6–8	3 d		0.03	0.5 (wet)	0.06	[85]
*Spirulina platensis, Chlorella vulgaris, Scenedesmus obliquus*	Ag NP	spherical,	8.2–20	28 ± 2		14 d	45	1	5	0.2	[90]
*Anabaena laxa*	Au NP	spherical, triangular, hexagonal, irregular	3–300	23 ± 1		14 d	25	0.1–1			[13]
*Euglena gracilis, Euglena intermedia*	Ag NP	spherical	6–60			12 h	27	1			[73]
*Coelastrella* sp., *Phormidium* sp.	Au NP	triangular, spherical	25–30			1 h	0	1	0.5	2	[94]
*Nostoc ellipsosporum*	Au NP	nanorod	137–209 (l), 33–69 (d)	20	4.5	2 d	20–30	0.04			[95]
*Leptolyngbya tenuis, Coleofasciculus chthonoplastes, Nostoc ellipsosporum*	Au NP	spherical, irregular, nanorods	8–30	20	5–9	5 d	20–30	0.003–0.3			[96]
*Stephanopyxis turris*	Au NP	spherical, triangular	10–30	18	8	5 h–8 d		0.0001–0.5			[82]
*Stephanopyxis turris*	Au-silica nanocomposite			18	8	2 d		0.2			[97]
*Chlamydomonas reinhardtii*	Ag NP	spherical	2–24	22 ± 1		1 d	69 ± 5	0.125–1.250			[69]
*Chlamydomonas reinhardtii*	Ag NP	irregular	n/a	22 ± 1		1 d	69 ± 5	0.125–1.250			[69]
*Chlorella kessleri, Dunaliella tertiolecta, Tetraselmis suecica*	Cu NP		15–65		6.8–7.7	72 h	50–230	0.5			[99]
*Chlorella vulgaris*	Ag NP		8–20	24 ± 2		3 d	54	1			[83]
*Tetraselmis kochinensis*	Au NP	spherical, triangular	5–35	28–29		2 d		1	10	0.1	[100]
*Phaeodactylum tricornutum*	Ag NP	spherical	<200	25		8–17 d	70	0–0.01			[101]
